# Authors' Reply

**DOI:** 10.1371/journal.pmed.0020095

**Published:** 2005-03-29

**Authors:** Tor B Stuge, Peter P Lee

**Affiliations:** Stanford UniversityStandford, CaliforniaUnited States of America

Drs. Speiser, Cerottini, and Romero [1] correctly point out that CD8+ T cells from HLA-A*0201 melanoma patients and healthy donors may contain small populations (on average, 0.07% ± 0.05% in their publications [2,3]) that bind tetramers made with the heteroclitic Melan-A M26 peptide, and that such cells express a naïve phenotype (CD45RA+). We too observe this phenomenon in a portion of HLA-A*0201 healthy donors and patients with melanoma that we analyze with M26 tetramers. Importantly, this is not seen in all subjects. These cells do not recognize the native M27 peptide, and represent cross-reactive subsets of naïve CD8+ T cells of multiple specificities [4]. We routinely analyze all subjects pre-vaccination, and the four post-vaccination responses analyzed in our report [5] did not contain M26 or gp100 tetramer-binding cells pre-vaccination (data not shown). Thus, it was unlikely that M26-cross-reactive cells spontaneously developed post-vaccination (not due to peptide vaccination) and were the basis of some of the low-recognition-efficiency MART-specific clones we analyzed. Furthermore, such a phenomenon has been seen only with M26 and not with the heteroclitic gp100 (G209-2M) peptide, so would not be a factor in the gp100-specific responses we analyzed.

The authors also point out that in their experience, they found that the majority of T cells generated with the heteroclitic Melan-A M26 peptide were tumor reactive, citing their studies in vitro [6], in mice [7], and in patients with melanoma [8]. We focus on their publication on patients with melanoma, as this is most directly relevant to our study. This report [8] focused on three patients with melanoma immunized with M26, and analyzed T cells from lymph nodes draining the vaccination site (vaccine-site sential nodes [VSSNs]). VSSNs from these three patients contained 0.11% (0.08%–0.15%) MART-specific T cells by tetramer staining. Importantly, contralateral lymph nodes in these subjects (distant from the vaccination site) also contained 0.06% (0.05%–0.09%) MART-specific T cells. With their reported background of less than 0.01%, this suggests the possibility of endogenous MART-specific T cells within lymph nodes in these subjects. These authors have shown in previous studies that endogenous MART-specific T cell responses frequently exist within lymph nodes, even in the absence of such cells in peripheral blood mononuclear cells [9]. Furthermore, these VSSN responses were analyzed only after two vaccinations, while the authors could not detect circulating MART-specific T cells in any of these three patients even after six vaccinations. MART-specific T cell lines were generated via tetramer-guided sorting of VSSN cells from patients 2 and 3, then individual clones generated via limiting dilution. They reported 16 of 17 clones killed A2+ MART+ melanoma targets. Without knowing the Vbeta usage of these clones and the Vbeta diversity of the parental MART-specific populations, it is difficult to know what fraction of each response these clones accounted for in the two subjects, as we have done in our study. More importantly, these tumor-reactive clones analyzed may be derived from endogenous T cell responses, possibly amplified by vaccination, rather than from de novo vaccine-elicited T cell responses. If so, these data would in fact fit well with our findings that endogenous responses consist mainly of cells with tumor-cytolytic potential that recognize the native peptide with high recognition efficiency.

In our study [5], we analyzed in detail four vaccine-elicited T cell responses (two to M26 and two to G209-2M) via the generation of more than 200 cytotoxic T lymphocyte clones, and assessed the fraction of each response that these clones accounted for collectively by analyzing the Vbeta usage of each clone and the parental peptide-specific populations. From this, we showed that the vaccine-elicited T cells were diverse in their tumor-cytolytic potential, which correlated with their recognition efficiency for the native peptides. It is important to point out that tumor-cytolytic T cells were present in these four subjects, but represented a significantly lower fraction than those derived from endogenous responses. These data are consistent with those we recently reported using a different experimental approach—assessing the fraction of T cells in a tetramer+ population that degranulate (via CD107 mobilization) to tumor stimulation [10]. While generating individual cytotoxic T lymphocyte clones and analyzing each for tumor killing and recognition efficiency, as we have done in this study, is the most definitive approach to analyze individual cells within an antigen-specific T cell response, this approach is extremely labor-intensive, and thus not feasible for large numbers of patients. As such, more rapid flow-cytometry-based methods, such as the CD107 mobilization assay and a new method to rapidly assess recognition efficiency of a T cell population via differential TCR downregulation (H. E. Kohrt, C. T. Shu, S. P. Holmes, J. S. Weber, P. P. L., et al., unpublished data), will allow analysis of many more patients to various vaccine formulations and strategies. This knowledge will be vital to the improvement of future cancer immunotherapies.
